# A Clinically Interpretable Computer-Vision Based Method for Quantifying Gait in Parkinson’s Disease

**DOI:** 10.3390/s21165437

**Published:** 2021-08-12

**Authors:** Samuel Rupprechter, Gareth Morinan, Yuwei Peng, Thomas Foltynie, Krista Sibley, Rimona S. Weil, Louise-Ann Leyland, Fahd Baig, Francesca Morgante, Ro’ee Gilron, Robert Wilt, Philip Starr, Robert A. Hauser, Jonathan O’Keeffe

**Affiliations:** 1 Machine Medicine Technologies Ltd., The Leather Market Unit 1.1.4, 11/13 Weston Street, London SE1 3ER, UK; sam@machinemedicine.com (S.R.); gareth@machinemedicine.com (G.M.); yuwei@machinemedicine.com (Y.P.); 2 Department of Clinical and Movement Neurosciences, Institute of Neurology, University College London, Queen Square, London WC1N 3BG, UK; t.foltynie@ucl.ac.uk (T.F.); krista.sibley.18@ucl.ac.uk (K.S.); 3 Dementia Research Center, Institute of Neurology, University College London, Queen Square, London WC1N 3AR, UK; r.weil@ucl.ac.uk (R.S.W.); l.leyland@ucl.ac.uk (L.-A.L.); 4 Neuroscience Research Centre, Molecular and Clinical Sciences Research Institute, St George’s, University of London, Cranmer Terrace, London SW17 0RE, UK; fbaig@sgul.ac.uk (F.B.); fmorgant@sgul.ac.uk (F.M.); 5 Department of Clinical and Experimental Medicine, University of Messina, Via Consolare Valeria, 98165 Messina, Italy; 6 The Starr Lab, University of California San Francisco, 513 Parnassus Ave, HSE-823, San Francisco, CA 94143, USA; roee.gilron@ucsf.edu (R.G.); robert.wilt@ucsf.edu (R.W.); philip.starr@ucsf.edu (P.S.); 7 Parkinson’s Disease and Movement Disorders Center, Department of Neurology, Parkinson Foundation Center of Excellence, University of South Florida, 4001 E Fletcher Ave, Tampa, FL 33613, USA; rhauser@usf.edu

**Keywords:** Parkinson’s disease, gait, time series analysis, computer vision, pose estimation, interpretable machine learning

## Abstract

Gait is a core motor function and is impaired in numerous neurological diseases, including Parkinson’s disease (PD). Treatment changes in PD are frequently driven by gait assessments in the clinic, commonly rated as part of the Movement Disorder Society (MDS) Unified PD Rating Scale (UPDRS) assessment (item 3.10). We proposed and evaluated a novel approach for estimating severity of gait impairment in Parkinson’s disease using a computer vision-based methodology. The system we developed can be used to obtain an estimate for a rating to catch potential errors, or to gain an initial rating in the absence of a trained clinician—for example, during remote home assessments. Videos (n=729) were collected as part of routine MDS-UPDRS gait assessments of Parkinson’s patients, and a deep learning library was used to extract body key-point coordinates for each frame. Data were recorded at five clinical sites using commercially available mobile phones or tablets, and had an associated severity rating from a trained clinician. Six features were calculated from time-series signals of the extracted key-points. These features characterized key aspects of the movement including speed (step frequency, estimated using a novel Gamma-Poisson Bayesian model), arm swing, postural control and smoothness (or roughness) of movement. An ordinal random forest classification model (with one class for each of the possible ratings) was trained and evaluated using 10-fold cross validation. Step frequency point estimates from the Bayesian model were highly correlated with manually labelled step frequencies of 606 video clips showing patients walking towards or away from the camera (Pearson’s r=0.80, p<0.001). Our classifier achieved a balanced accuracy of 50% (chance = 25%). Estimated UPDRS ratings were within one of the clinicians’ ratings in 95% of cases. There was a significant correlation between clinician labels and model estimates (Spearman’s ρ=0.52, p<0.001). We show how the interpretability of the feature values could be used by clinicians to support their decision-making and provide insight into the model’s objective UPDRS rating estimation. The severity of gait impairment in Parkinson’s disease can be estimated using a single patient video, recorded using a consumer mobile device and within standard clinical settings; i.e., videos were recorded in various hospital hallways and offices rather than gait laboratories. This approach can support clinicians during routine assessments by providing an objective rating (or second opinion), and has the potential to be used for remote home assessments, which would allow for more frequent monitoring.

## 1. Introduction

### 1.1. Parkinsonian Gait

Walking is critical to independent mobility, activities of daily living and quality of life [1], and can be affected by a large number of factors including age, sex [2], height [3], weight [4] and emotional state [5]. Gait is commonly impaired in numerous neurological diseases [6], including Parkinson’s disease (PD) in which it is progressively impaired and ultimately becomes a key source of disability [7].

Gait impairments in PD are complex and symptoms vary across individuals but commonly include a reduction in velocity, shorter stride length, reduced arm swing, involuntary limb posturing (dystonia) and a stooped posture [8]. As the disease progresses, additional symptoms such as freezing of gait, dyskinesias and balance impairment become more common [7].

In clinical practice, the assessment of PD is commonly performed based on the Movement Disorder Society Unified PD Rating Scale (MDS-UPDRS, [9]), wherein gait is assessed using a combination of patients’ verbal accounts of their daily living (items 2.12 [Walking and Balance] and 2.13 [Freezing]) and rater evaluations (items 3.10 [Gait] and 3.11 [Freezing of Gait]). For the rater assessment of gait (item 3.10), the patient is asked to walk away from and towards the examiner who then estimates a severity score on a 5-point scale between “normal” and “severely impaired” for this action (see Supplement for additional details about the instructions). Although assessors are usually highly trained and the score categories are made as clear as practicable, at least, to some extent, they are subjective, and it is not uncommon for raters to diverge from one another by one point [10].

### 1.2. Technology

Technological advances have made it possible to obtain a rich characterisation of gait by using specialised equipment and/or dedicated laboratories [11,12,13]. Previous studies have relied on a variety of technologies, including gait walkways and wearable sensors such as accelerometers or rhythmograms, for objective assessments of gait in PD. Although they allow for a detailed characterisation of gait, they are typically burdensome to both subjects and assessors, requiring additional equipment, and adding complexity, time and cost to the assessment [14,15,16], as well as often being impracticable in the home environment.

However, it is already common practice for clinicians to record video during gait examinations using commercially available camera equipment. In combination with recent advances in deep learning based markerless pose estimation [17,18,19,20,21], this allows for an algorithmic system to objectively measure features of a patient’s gait from a video recording, and then estimate an objective severity score. Such a system could be employed without requiring additional equipment, cost, or inconvenience for examiners or patients.

### 1.3. Previous Work

A recent review of video gait analysis found the majority of research focused on marker based pose estimation, noting that such systems can result in error due to inconsistent marker placement, with only a small number of studies (3 of 30) using markerless pose estimation [22].

Previous work has used markerless pose estimation for gait analysis [23], but only a small portion of studies involved patient populations [22]. The existing studies including PD patients had low sample sizes, meaning models were trained using many video clips of the same few patients, while the MDS-UPDRS ratings used as ground truth were made by a single clinical assessor [24,25]. This means the models were trained to agree with a single clinician, and could only learn about the small set of PD manifestations seen in a few patients.

Studies focusing on classification of PD gait achieved good accuracy, but relied on “black box” systems which are difficult to interpret [25]. Previous studies focusing on interpretability of gait analysis systems used marker based motion capture systems [26,27], and devices such as inertial measurement units [28]. However, to our knowledge, no study has focused on interpretability of systems that utilise markerless pose estimation for classification of PD gait.

### 1.4. Our Approach

A robust system for quantifying Parkinsonian gait requires a dataset consisting of many patients, in order to learn the large number of ways in which the condition can manifest, with clinical ratings made by many different assessors, in order to deal with the subjectivity of MDS-UPDRS ratings.

We proposed and evaluated a novel approach for estimating severity of gait impairment in Parkinson’s disease using a computer vision-based method, utilising a dataset consisting of hundreds of patients, examined at different sites by thirteen assessors. This is the first study to validate that computer vision methods to classify Parkinsonian gait can learn from the opinions of many clinical assessors and generalise across a wide patient population.

The system used markerless pose estimation to extract objective features of patients’ gait characteristics, which were then used to train a machine learning model to estimate a severity score. We showed how objective estimates of features and labels could be used to support clinicians’ decision-making.

Extracted features and model estimates are closely linked to key aspects of gait, making them easily interpretable. This also allows clinicians to understand which characteristics of a patient’s gait caused the model to estimate a certain score, and why the model’s rating might differ from their own estimate.

As it is already common to record videos of PD assessments, our approach seamlessly integrates into existing clinical practice. It would offer clinicians a second objective opinion for MDS-UPDRS ratings, and could be used to gain an initial rating in the absence of a trained examiner—for example, during remote home assessments.

## 2. Materials and Methods

### 2.1. Proposed Methodology

**Figure 1:**
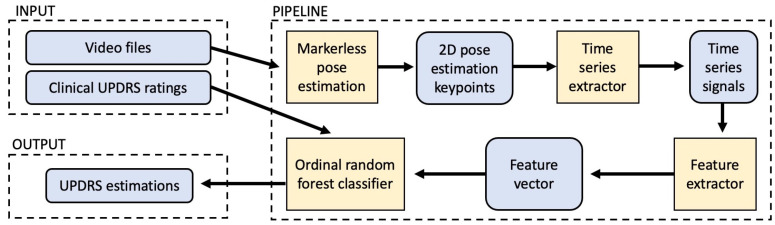
An overview of the pipeline used for this study, which included markerless pose estimation, signal estimation, feature extraction and classification.

Our methodology is summarised in Figure 1, which shows the end-to-end pipeline of the computer-vision system. Inputs are videos and UPDRS ratings from clinics (Section 2.2). These feed into a pipeline which begins with markerless body key-point detection, with sequential key-point coordinates used to construct time-series signals which characterise gait (Section 2.3). From these signals, features are extracted which are designed to capture the gait characteristics of arm swing, roughness of walking, postural control Section 2.5) and speed (Section 2.4). Finally, these features feed into an ordinal classification model (Section 2.6), which outputs estimates of UPDRS ratings.

### 2.2. Data

**Figure 2:**
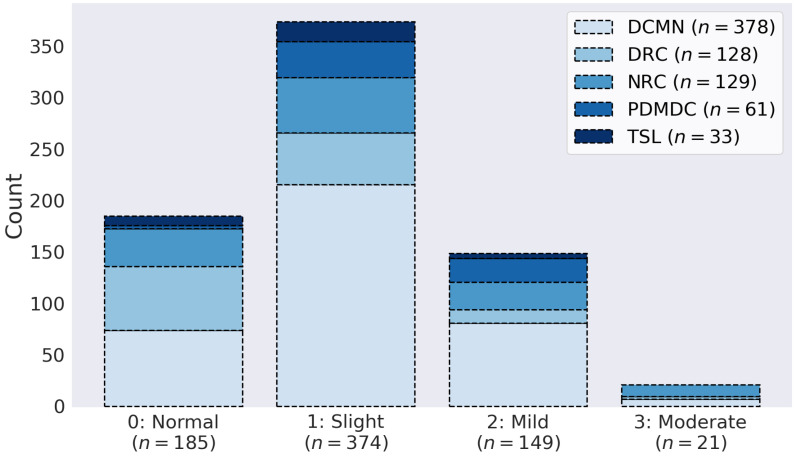
The distribution of UPDRS ratings across the five assessment centres. Severity scores were imbalanced, with low scores being more common than high scores, reflecting the distribution of ratings commonly encountered in the clinic [30]. DCMN: Department of Clinical and Movement Neurosciences, Institute of Neurology, University College London; DRC: Dementia Research Center, Institute of Neurology, University College London; NRC: Neuroscience Research Centre, Molecular and Clinical Sciences Research Institute, St. George’s, University of London; PDMDC: Parkinson’s Disease and Movement Disorders Center, Baylor College of Medicine; TSL: The Starr Lab, University of California San Francisco.

Videos were recorded using the KELVIN-PD™ mobile application and then collected on the KELVIN-PD™ motor assessment platform developed by Machine Medicine Technologies [29]. Examiners included nurses, neurologists and researchers who performed UPDRS assessments of PD patients at one of the five largest sites currently using this platform (Figure 2). Gait (impairment) was rated on a 5-point ordinal scale ranging from “normal” to “severe” (ref. [9], see also Supplement for details about the MDS-UPDRS instructions).

We analysed 729 videos showing gait assessments (“item 3.10”) of patients who received a score of 0–3. The recordings show patients walking directly towards and/or away from the camera. Approximately two thirds (481/729, 66%) of the videos showed examinations of patients who had recently taken PD medication. Many of the videos were recorded as part of `levodopa challenges’, whereby patients are assessed before taking levodopa and again after taking it (252 videos, corresponding to 126 levodopa challenges). Two videos showing “severity 4” (i.e., severe impairment, usually meaning patients are unable to walk) were not included. Importantly, we did not perform any manual selection of videos, and they therefore accurately reflected the current state of data routinely collected at these institutions. The videos were recorded by a variety of different assessors, using different cameras (integrated within their mobile device), in hallways or office settings.

We manually annotated all videos with regions of interest (ROIs) of times when patients were walking towards or away from the camera, without including the section of the video when they were turning. All videos except one showed both directions and we therefore extracted two ROIs from them. In addition, we counted the number of steps during a subset of 606 ROIs (302 “away”, 304 “towards”) and used this information together with the length of the ROIs to calculate the “ground truth” step frequency for these video clips. The mean length of all ROIs was 210 frames (approximately 7 s at 30 frames per second).

### 2.3. Signals

**Figure 3:**
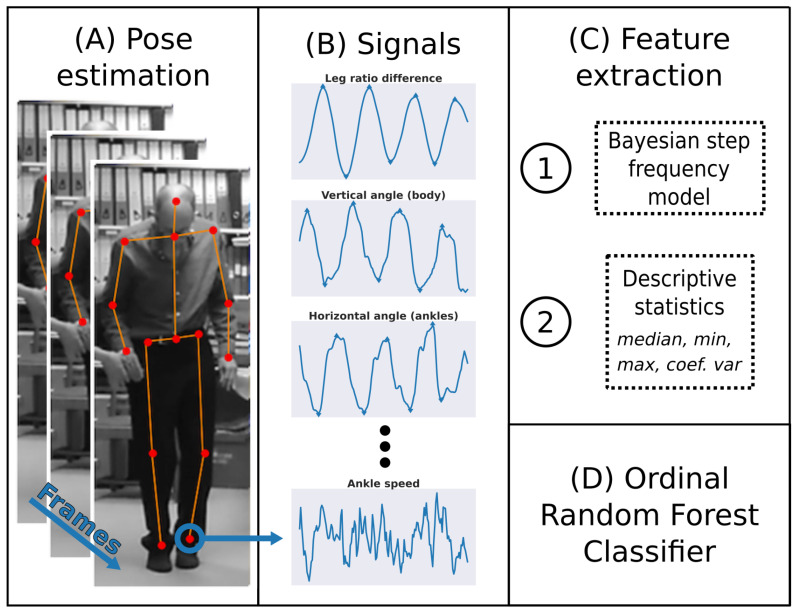
Methods overview. (**A**) Body key-points were extracted from each frame using the deep learning library OpenPose [17]. (**B**) Signals were created by combining the time-series of various key-points (see Section 2.3 and Table 1). (**C**) Features were extracted from the signals based on two different methods (Section 2.5): (1) a Bayesian step frequency model integrating information from three signals over time, and (2) summary statistics such as the median amplitude. (**D**) An ordinal random forest classifier was used to estimate patients’ UPDRS scores (see Section 2.6).

The deep learning library OpenPose [17] was used to extract 25 body key-point coordinates on each frame without any markers (Figure 3A). OpenPose is a popular open-source library (The GitHub repository (https://github.com/CMU-Perceptual-Computing-Lab/openpose (accessed on 16 July 2021)) has more than 20,000 stars and more than 6000 forks) providing state-of-the-art pose estimation performance. Sequential key-point coordinates were used to construct seven normalised time-series signals as follows.

The *leg ratio difference* ($R_{legs}$) was defined as the difference between the ratios of left-to-right and right-to-left leg lengths. The *vertical angle of the body* ($A_{body}^{\left[vert\right]}$) was defined as the angle between the *y*-axis of the video and the line going through the neck key-point and the mid-point between the two ankle key-points. The *horizontal angle of the ankles* ($A_{ankles}^{\left[horiz\right]}$) was defined as the angle between the *x*-axis of the video and the line going through the two ankle key-points. Similarly, the *horizontal angle of the wrists* ($A_{wrists}^{\left[horiz\right]}$) was defined as the angle between the *x*-axis of the video and the line going through the two wrist key-points. The *horizontal distance between the heels* ($D_{heels}^{\left[horiz\right]}$) was defined as the distance between the *x*-coordinates of the two heel key-points, normalised by the estimated standing height of the patient. Finally, the *speed of the left (and right) ankle* ($D_{ankle(L)}^{\left[Eucl\right]}$ and $D_{ankle(R)}^{\left[Eucl\right]}$) was defined as the Euclidean distance between coordinates of the left (and right) ankle on successive frames, normalised by the estimated standing height. Table 1 summarises these signals and their equations.

A peak detection algorithm (see Supplement) was used to extract “peaks” and “troughs” from the four signals $R_{legs}$, $A_{body}^{\left[vert\right]}$, $A_{ankles}^{\left[horiz\right]}$ and $A_{wrists}^{\left[horiz\right]}$. By definition, peaks and troughs of the leg ratio difference signal correspond to a maximal difference between left and right leg; i.e., they were expected to be detected on frames at the end of each gait cycle. Similarly, periodically occurring peak and trough “events” of the other signals were expected to reflect gait cycles.

### 2.4. Step Frequency (Speed)

**Figure 4:**
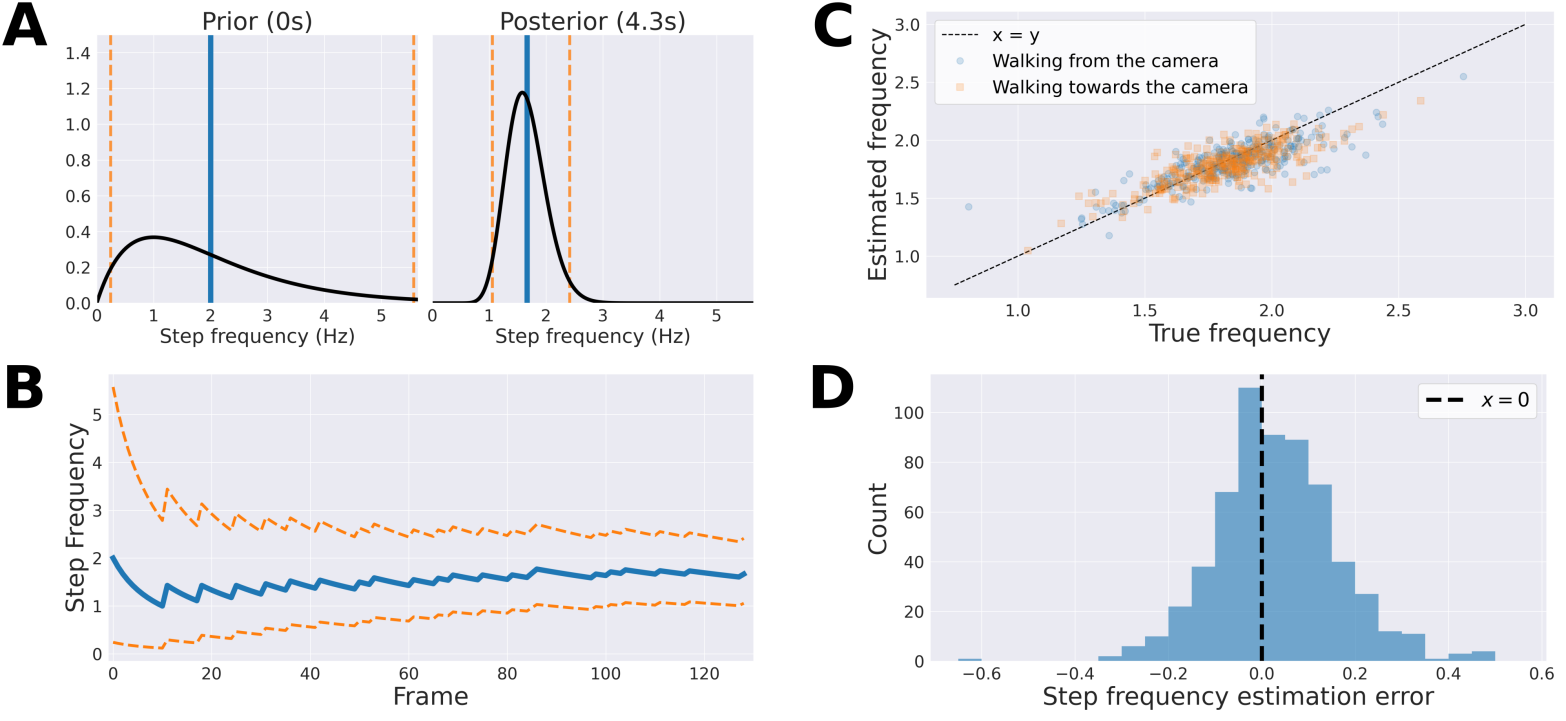
Bayesian step frequency estimation. (**A**) Examples of the prior distribution (left) and a posterior distribution after 129 updates (i.e., after 129 frames or approximately 4.3 s of video); (**B**) the evolution of the posterior mean and 95% credible interval for the first 129 updates; (**C**) point estimates of the Bayesian step frequency model’s posterior distributions at the last frame of each video were highly correlated with the true labels (Pearson’s r=0.80, p<0.001). The mean squared error between estimated and true step frequency was 0.018Hz; (**D**) the distribution of errors of step frequency point estimates in the last frame of each video. The mean error was 0.03Hz, indicating a tendency to under-predict. The null-hypothesis that the population is normally distributed was rejected (Shapiro Wilk’s W=0.98, p<0.001).

Step frequency (speed) is known to be an important characteristic of gait [8] and is generally altered in PD [7]. Here, step frequency was estimated using the three signals $R_{legs}$, $A_{body}^{\left[vert\right]}$ and $A_{ankles}^{\left[horiz\right]}$. A posterior distribution over step frequency was obtained for each frame using a Gamma-Poisson model (see also Figure 4A,B):

(1) $$\lambda ∼\mathrm{Gamma}(\alpha_{0}+\sum_{i=1}^{N}Y_{i},\beta_{0}+N),$$

where $\alpha_{0}$ and $\beta_{0}$ are the parameters for the prior; $Y_{i}$ is the number of events across the three signals in the *i*th frame, and *N* is the number of elapsed time intervals (i.e., three times the length of the ROI divided by the frame rate of the video).

The prior was set to $\lambda ∼\mathrm{Gamma}(\alpha_{0}=2,\beta_{0}=1)$, which corresponds to a distribution with a 95% credible interval of (0.24, 5.57) Hz, and for which the mean is $E\left[\lambda \right]=\alpha_{0}/\beta_{0}=2$. The choice of prior reflects the range of plausible human movement. A step frequency of 2Hz is typical of normal gait, 5.57Hz is achievable by talented sprinters, and movement slower than 0.24Hz no longer resembles a continuous gait cycle.

The posterior was updated at each frame as

(2) $$\mathrm{Gamma}(\alpha_{i}=\alpha_{i-1}+Y_{i},\beta_{i}=\beta_{i-1}+\frac{i\times 3}{F}),$$

where *F* is the frame rate of the video. The final step frequency estimation for the video was the mean of the posterior ($E\left[\lambda \right]=\alpha_{k}/\beta_{k}$) at frame *k* during which the last event occurred.

Performance of the model was evaluated by calculating the mean squared error of the estimation in relation to the manually labelled ground truth step frequencies.

### 2.5. Features

In addition to speed, five other features were extracted from the signals described in Section 2.3. These features were chosen because we hypothesise that the values of these features would covary with clinical judgements of disease severity. We furthermore confirmed the clinical relevance of our features by showing that they correlate with severity ratings and are affected by medication (see Section 3).

Two features related to patients’ “arm swing” were extracted from the horizontal angle of the wrists ($A_{wrists}^{\left[horiz\right]}$) time-series signal: (a) the median of the absolute first difference of the signal, (“median velocity”), and (b) the median amplitude at its peaks for which we used the detected troughs to span a “lower bound” and then computed the height of the signal from this lower bound at each peak, which were then averaged. Arm swing is an important characteristic of human gait [31] and is commonly reduced in PD [32], often early in the disease progression [33,34]. The MDS-UPDRS instructions [9] list arm swing as one of the rating criteria for gait.

Two features were used to capture patients’ roughness of walking. They were based on the speed of the left and right ankle ($D_{ankle}^{\left[Eucl\right]}$). These features were calculated as the median of the first difference of this signal (“absolute acceleration”) divided by the value of the $D_{ankle}^{\left[Eucl\right]}$ signal on each frame. For classification, we re-coded these features as minimum and maximum feature values instead, to make them independent from laterality. A number of studies used accelerometers to measure (ankle) acceleration and showed that it can contain important information about PD severity [35,36]. Beck et al. [37] used acceleration data to estimate smoothness in gait, and found that smoothness measures were lower in PD patients than healthy controls. For our features, a less smooth movement should result in higher “roughness” feature values than very smooth movement (see Supplement for additional details).

The horizontal distance between the heels ($D_{heels}^{\left[horiz\right]}$) was used to estimate patients’ variability in the width of their strides. A feature was calculated as the coefficient of variation of the whole signal, and was used as a measure of postural control. Postural control is an important factor for the assessment of quality of gait in older adults [38] and Parkinson’s disease [39]. A recent study applied machine learning techniques to distinguish between PD patients and healthy controls, and concluded that stride width variability was one of the most important features for the classification of these groups [40]. Similar to our results (Section 3.1), they found that healthy participants had a higher stride width variability than PD patients, indicating lower postural control in PD.

### 2.6. Classification

An ordinal classifier [41], based on random forest classifiers (RFCs), was trained and evaluated using 10-fold (stratified) cross validation. Ordinal classification was used because classes (degrees of impairment) are inherently ordered. The ordinal classification system was internally comprised of three binary RFCs which were trained to distinguish {0} vs. {1,2,3}, {0,1} vs. {2,3} and {0,1,2} vs. {3}. For example, the probability of class {1} can then be computed as the probability of classes {0,1} (from classifier 2) minus the probability of class {0} (from classifier 1). Due to the class imbalance (Figure 2), we used the Synthetic Minority Oversampling Technique (SMOTE; [42]) to up-sample minority classes within each training fold. Multiple ROIs per video (“towards” and “away from” camera) were treated as independent samples within each training fold. Within testing folds, the classifier made a prediction for each ROI (i.e., estimated a probability for each of the four classes), which were then averaged to give the prediction for the video. Importantly, both ROIs of each video were ensured to always be in the same (training or testing) fold to avoid information leakage.

We trained and evaluated six types of models: Random Forest Classifier, Linear Discriminant Analysis, Logistic Regression, Artificial Neural Network, Linear Support Vector Machine, and Gradient Boosted Trees. Details about these models are shown in the Supplement. Each model was used as a based classifiers within an ordinal classification system as described above. The RFC was chosen because it achieved the highest accuracy, although overall all six ordinal classifiers achieved similar performance (see Section 3).

Primary metrics used to judge the model’s performance were balanced accuracy (mean of correct proportion per class), *accuracy* (±1), binary sensitivity and binary specificity. The *accuracy* (±1) metric was defined as the proportion of estimates for which the absolute residuals were one or less, and was used because it is not uncommon for UPDRS assessors to disagree with one another by one point [10]. For the binary metrics, all Parkinsonian ratings ({1,2,3}) were grouped together as the “positive” class, and the non-Parkinsonian rating ({0}) was denoted the “negative” class.

### 2.7. Explainability and Interpretability

In addition to relying on well defined and interpretable computations of features in general, we also focused on the explainability of specific model estimates. Especially in clinical applications, if a model is to be used to support a clinician’s decision-making, it can be important to understand (a) what a specific feature value means and how it is related to feature values of other patients, and (b) how a model arrived at its decision.

Given the objectively estimated feature values of some specific sample, we calculated where in the distribution of all examples in our data set the specific feature value would fall. For a specific feature value, *v*, we computed the proportion of feature values smaller or equal to *v*, conditioned on each severity. More precisely, for a video with estimated feature values $\left\{v_{1},\ldots ,v_{6}\right\}$, we calculated an “eccentricity table” (or matrix), *C*, so that, for each entry,

(3) $$C_{i,s}=\frac{1}{N_{s}}\sum_{j}^{N_{s}}I\left(v_{i},v_{i}^{\left(j\right)}\right),$$

where $v_{i}^{\left(j\right)}\in D_{s}$ is the value of feature *i* for sample *j*, $D_{s}$ is the set of samples with a severity rating *s*, $N_{s}$ is the cardinality of $D_{s}$, and

(4) $$I\left(x,y\right)\left\{\begin{array}{cc} 1 & \mathrm{if}\;x\geq y\\ 0 & \mathrm{otherwise}. \end{array}\right.$$

To ease interpretation, we shaded the tables according to absolute distance from the center of the distribution, meaning values close to the center of the distribution would receive a darker shade. For a “typical” patient receiving a rating of 0, it would be expected that the first column (which corresponds to typical distributions for severity 0), showing the data conditioned on severity 0, would be the darkest. We provided examples which show how these eccentricity tables could be used to support a clinician’s decision-making.

SHAP (SHapley Additive exPlanations) values [43,44] can be used to understand why a model made a certain prediction. For a specific example, more important features, i.e., features which contributed more to the prediction for this example, receive larger (absolute) SHAP values. Because our ordinal classifier was comprised of three binary random forest classifiers, we could compute SHAP values for each of these three classifiers. We show how SHAP values, in combination with eccentricity tables, could provide valuable “step-by-step” insight into how the model arrived at its severity estimate.

## 3. Results

### 3.1. Objective Feature Values

Manually labelled and automatically estimated step frequencies were highly correlated (Pearson’s r=0.80, p<0.001; Figure 4). The mean squared error (MSE) between estimated and ground truth step frequencies was 0.018Hz, and was similar between clips showing patients walking from (MSE =0.019Hz) or towards (MSE =0.017Hz) the camera. At the end of the video clips, ground truth step frequency fell within the 95% credible interval of the posterior distribution in 605 of 606 cases (99.8%). Step frequency estimates were significantly higher for patients with non-Parkinsonian compared to patients with Parkinsonian gait ratings (Welch’s t(827.5)=9.43, $p<10^{-10}$).

**Figure 5:**
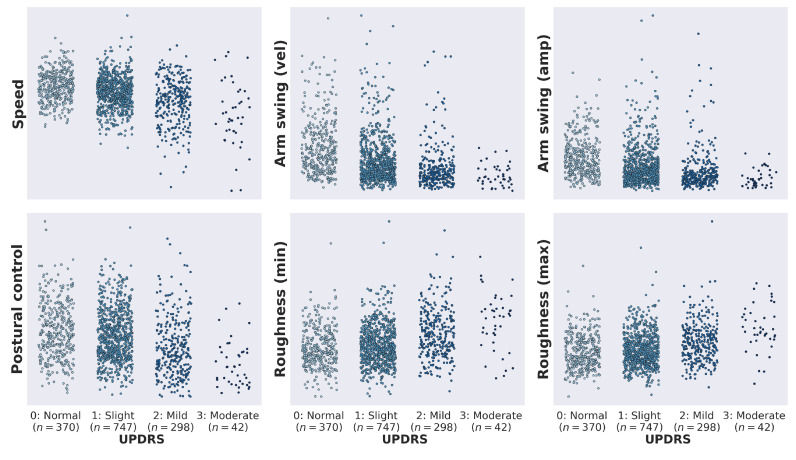
Distribution of the six features by clinical UPDRS gait (item 3.10) rating. For each of the six features, a one-way ANOVA test found a highly significant (p<0.001) difference in means between the clinical UPDRS groups. All features were significantly correlated with total UPDRS part-III scores (see Table 2 and Figure 6).

**Figure 6:**
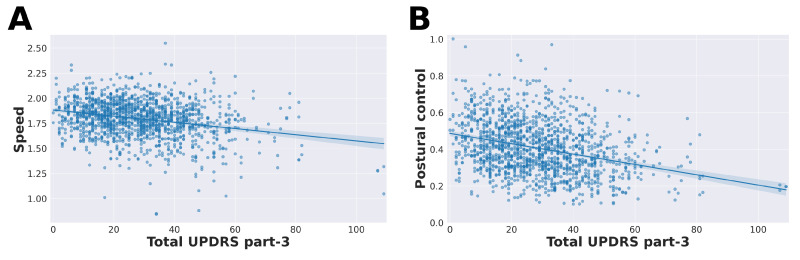
Correlation of feature values with total UPDRS part-III scores. Most features were significantly correlated with total UPDRS part-III scores (Table 2). (**A**) Estimated step frequency (speed) was significantly correlated with total UPDRS part-III scores (Pearson’s r=−0.26, p<0.001). (**B**) Postural control feature values were significantly correlated with total UPDRS part-III scores (Pearson’s r=−0.31, p<0.001).

Figure 5 shows the distribution of all feature values conditioned on UPDRS ratings. We also looked at the association between estimated feature values and total MDS-UPDRS part-III scores which include 18 items [9]. All features were significantly correlated with total UPDRS part-III scores (see Table 2 and Figure 6), indicating that they are related to general disease progression.

**Figure 7:**
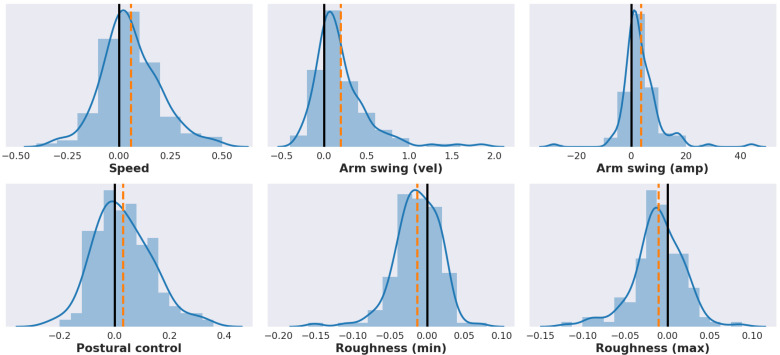
Distributions showing the change in each feature value related to medication (n=126, in each subplot), with the mean value marked as an orange dashed line. The change is calculated as the feature value during the `on medication’ assessment, of the levodopa challenge, minus the `off medication’ assessment. As the medication generally improves motor function for PD patients, the directions of change make sense intuitively (see also Figure 5). For example, feature values related to speed and arm swing increased after taking medication. For most features, the change in feature value was significant (see Table 3).

An effect of medication was evident within the 126 levodopa challenges in our data set. We looked at the differences of estimated feature values in assessments conducted after and before the patient took their medication (“on medication” minus “off medication”), see Figure 7). These differences were significant for 5 of the 6 features (Table 3).

### 3.2. Model Comparison

All six ordinal classifiers achieved similar performance (Table 4). The RFC achieved the highest accuracy, *accuracy* (±1) and Spearman’s correlation. The linear SVM achieved the highest balanced accuracy. The random forest was chosen as the primary classifier and the following results are based on that model. The supplement includes additional details about results based on other models.

### 3.3. Model Performance

**Figure 8:**
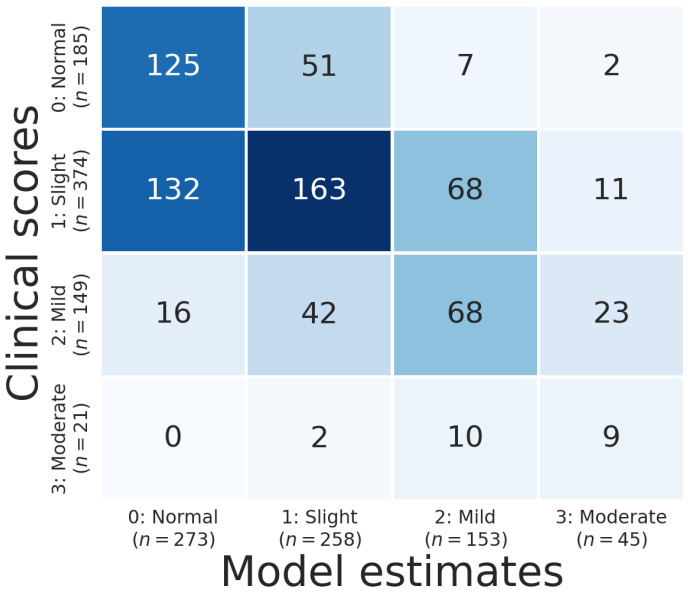
Confusion matrix showing the results from the 10-fold cross-validation based on ratings given by the original examiners at the clinical sites where the assessments were performed. Note that 15 videos were later re-rated by a senior neurologist, which changed the ratings of six videos (see Table 5).

Figure 8 shows the confusion matrix of the model estimates. Balanced accuracy was 50%, which outperformed chance significantly (two-tailed label permutation-based [46], p<0.001). *accuracy* (±1) was 95% (*accuracy* (±2) was 99.7%), binary sensitivity was 73% and binary specificity was 68%. This means our model greatly outperformed chance performance and diverged from clinicians’ ratings by more than one point in only 5% of cases. There was a significant correlation between clinician labels and model estimates (Spearman’s ρ=0.52, p<0.001). In addition, 125 of 185 patients were correctly identified as healthy walkers, and 396 of 544 patients with a Parkinsonian gait rating were correctly identified as displaying symptoms of an impaired gait.

### 3.4. Interpretability of the Model Features

**Figure 9:**
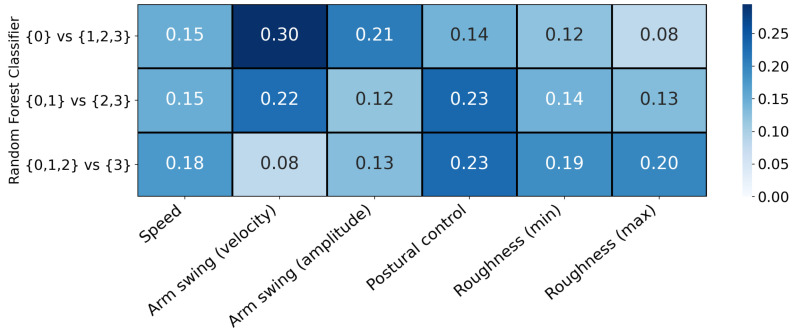
Feature importance of the three random forest classifiers contained within the ordinal classifier. The impurity-based (Gini) importance was calculated as the normalized total reduction of the Gini coefficient by the feature. Arm swing features were important to distinguish normal gait from Parkinsonian gait. Roughness of movement features were important to distinguish between different levels of Parkinsonian impairment.

We inspected the feature importance for each of the three RFCs contained within the ordinal classifier trained on the full data set (Figure 9). The impurity-based (Gini) importance was calculated as the normalized total reduction of the Gini coefficient [47] by the feature [48]. (See Supplement for the ranking of feature importance based on SHAP values, which was almost identical.) Consistent with previous reports [33,34], arm swing was found to be important for distinguishing normal gait from Parkinsonian gait but became less important for the classification in later stages of the disease. Roughness of movement was found to be relatively less important for detecting Parkinsonian gait in general but very important for distinguishing between higher UPDRS ratings. This is consistent with reports by Rastegari et al. [49] who found that data from ankle accelerometers could be used to distinguish between healthy controls and later-stage PD patients, but not between healthy controls and early-stage PD patients. Similarly, Hatanaka et al. [50] reported significantly different mean acceleration between healthy control and PD patients, with the difference being more pronounced in later stages of the disease.

### 3.5. Interpretability of Model Estimates

**Figure 10:**
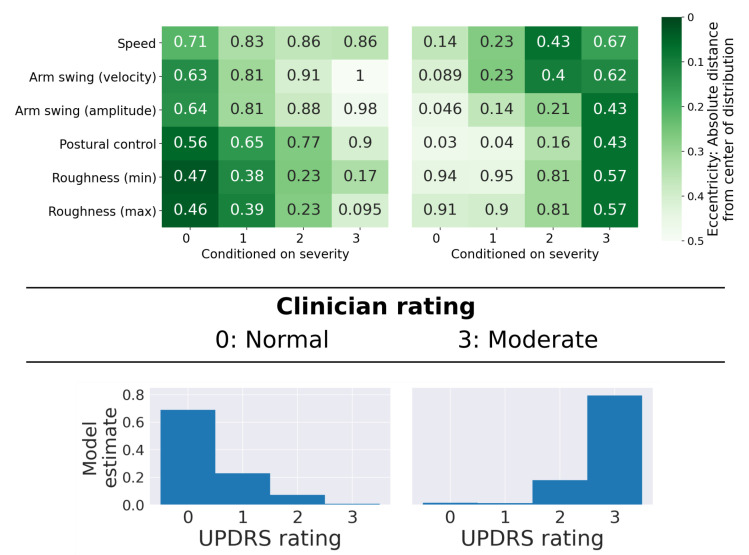
Examples with complete agreement between clinician and model. At the top, eccentricity tables for patients who were rated as “normal” (**left**) and “moderately impaired” (**right**) are shown. At the bottom, the model’s estimates for the two examples are shown. In both cases, the model agreed with the clinician and estimated the correct ratings with high probability.

Figure 10 shows two examples for which clinician and model ratings agreed. In both cases, the eccentricity tables illustrate that feature values are typical of the scores they received. For a clinician, this can provide valuable supporting evidence that their rating is likely accurate.

**Figure 11:**
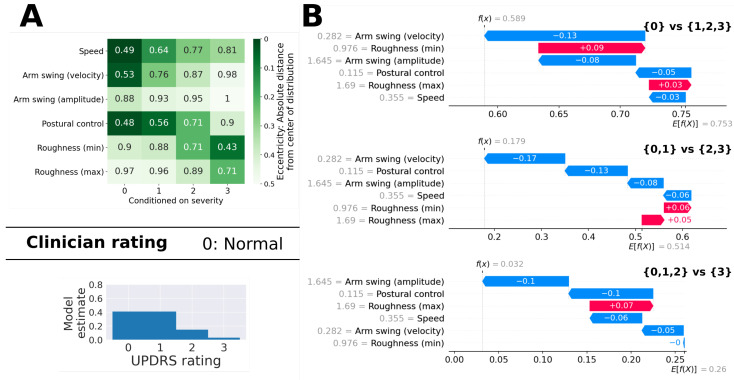
Example with a slight disagreement between the clinician’s rating and the model’s estimate. (**A**) The eccentricity table at the top shows a less clear structure than the example in Figure 10. While speed, arm swing, and postural control are typical of low severity ratings, the roughness of the movement was fairly typical for higher severity ratings. The Clinician gave the patient a rating of 0, while the model estimated a score of 1, although the distribution at the bottom shows that the model’s probability estimate for rating 0 was very close to the probability for rating 1. (**B**) The three figures illustrate how the model arrived at its estimates. We computed SHAP values for the example based on each of the three different binary classifiers which are part of the ordinal model. It can be seen that, in all three classifiers, all feature values “push” towards lower ratings, except the two feature values that are related to the roughness of movement. The first classifier estimated the probability of the example receiving a rating of greater than 0 as 59%. The second classifier estimated the probability of the example receiving a rating of 2 or 3 as 18%. For both of the first two classifiers, the specific value for the “arm swing (velocity)” feature was most important.

Figure 11A shows an example for which there was a slight disagreement between the clinician (rating 0) and the model (rating 1, although class 0 had an almost identical probability). The eccentricity table shows that, while most feature values (speed, arm swing, postural control) were typical of patients with a rating of 0, the roughness of movement feature values were more typical of more severe ratings. Figure 11B provides a more detailed explanation of how the ordinal classifier arrived at its probability estimates. Within the first internal classifier (which distinguishes between {0} and {1,2,3}), the roughness features were deemed important enough to cancel out most of the “push” of the other features towards a low rating. Within the second classifier, the feature values for roughness were considered less important (i.e., they added less to the prediction) and the model estimated a probability of only 18% for ratings {2,3}. Note that the probability for rating 1 (41.1%) is computed as the probability of ratings {0,1} (82.1%) minus the probability of rating 0 (41%).

### 3.6. UPDRS Score Re-Ratings

We asked a senior neurologist (Prof. Thomas Foltynie, UCL Queen Square Institute of Neurology) for his expert opinion about 15 videos. Five videos were selected for which the model disagreed with the original rater by 2 points. Four videos with a disagreement of 1 and six videos with a disagreement of 0 were also randomly selected. The expert was asked to provide a rating for all of these videos without any information about the original assessor’s or the model’s scores. Table 5 shows the results of this re-rating. For the five videos with an original score difference of 2, the expert agreed with the original ratings in only a single case. Three videos were re-rated with a score in between the original examiner’s and the model’s score, and, in one case, the expert disagreed with the original rating by two points which matched the model estimation. For the four videos with a rating difference of one, the expert agreed with the original rater in all cases. For four of the six videos with zero difference, the expert agreed with model and clinician ratings, and, in two cases, disagreed with them by one point. See Supplement for additional analysis (eccentricity tables and SHAP values) of some of these examples.

## 4. Discussion

### 4.1. Overview of Results

Video data of gait assessments were collected at five active clinical sites. The collection was part of the routine examinations of Parkinson patients and did not require additional equipment or time. Markerless pose estimation was used to extract objective features characterizing patients’ gait. Features included step frequency which was estimated based on a Bayesian model. Comparison with ground truth showed that step frequency point estimates were highly accurate. An ordinal random forest classifier was trained to estimate UPDRS severity scores. It achieved high performance (50% balanced accuracy) and only rarely diverged from clinical examiners’ ratings by more than one (95% *accuracy* (±1)).

By sending fifteen videos for re-rating, we showed that ratings by clinical assessors can often disagree. Given that our model was trained on ratings from multiple assessors, and so has the benefit of learning from multiple perspectives, it is possible that the model could outperform any individual assessor. We also provided examples of how a clinician could understand objective feature and model estimates and how this could support decision making.

Although much work remains to be done before gait severity scores can be reliably estimated completely autonomously, useful applications in quality control are already plausible. Our results suggest that any disagreement between the examiner and model’s estimation would in approximately 5% of cases be large (rating of 2 or more points) and in roughly 50% cases be small (rating of 1 point). This means the model could, for example, be used to make central ratings far more efficient by identifying the subset of data points which are likely to require re-rating, thus eliminating the need to re-rate all samples. Objective model estimates could be used to improve clinical ratings, and, as mistakes are discovered and corrected through re-ratings, model performance would also be expected to improve, resulting in a beneficial cycle of improvements and standardisation.

### 4.2. Comparison with Previous Work

As far as we are aware, only a single group, Lu et al. [25], has tried to tackle the task of estimating UPDRS scores of PD patients directly from video data. They used a neural network classifier to estimate severity within a small group of 30 PD patients, who were all assessed by a single rater, and achieved a balanced accuracy of 81%. Notably, although the accuracy in the current study is lower, it is based on a much larger sample collected at multiple clinical sites by multiple raters and our results are therefore much more likely to generalise. In addition, while neural networks are powerful, they are also a “black-box” approach and feature interpretation is difficult.

One other group, Sabo et al. [24], estimated UPDRS scores from video data, although the patients were not diagnosed with PD. While this research did provide clinically interpretable features, the work used a much smaller sample size, with multiple recordings of each patient. This means it is less likely to generalise (to the wider PD patient population) than our result.

Previous studies have used a range of different technologies, including accelerometers [51,52], load sensors integrated into shoes [53,54,55,56], inertial measurement units [57], gyroscopes [12], and pressure-sensitive walkways or other laboratory setups [13] to objectively capture gait parameters. Due to their fixed place and high cost, dedicated gait laboratories are unlikely to be useful for routine assessments during clinical practice.

Although wearable technologies provide some advantages as they allow patients to move freely and can be employed at any location, they still require additional dedicated equipment which needs to be bought and maintained. In addition, while such wearable technologies have the potential to provide more precise measurements, in comparison to video data, issues around set up, such as inconsistent marker placement in motion capture systems, can result in errors [22].

In contrast to these technologies, video data are already routinely collected at many institutions (including the five sites in this study) and therefore do not require any additional equipment. It can be collected on any smart phone or tablet with integrated video recording capability and does not add time or cost to the gait examinations.

### 4.3. Interpretability of Results

Our method provided transparent and clinically interpretable computations. The Bayesian step frequency model relied on three signals, and detected peaks and troughs within these signals, extracted from each video clip, and updated its prediction on each frame. Because a continuous probability distribution was maintained over step frequency, point estimates were accompanied by measures of uncertainty. Step frequency estimates were robust for both patients walking towards and away from the camera, and, for relatively short video clips (mean duration of 7 s), thereby increasing flexibility for assessors. Consistent with previous work [58], step frequency (speed) was significantly reduced in Parkinsonian gait (UPDRS rating ∈{1,2,3}), compared to “normal” gait (rating 0).

Other features were constructed to measure arm swing, postural control, and roughness of movement. Many of these have been examined in previous studies and are generally altered in PD [7]. All features showed a strong association with total UPDRS part-III scores, indicating that objectively calculated features based on patients’ gaits might be useful for tracking general disease progression. A reduction in arm swing can be seen early in the disease progression [33,34], while changes in acceleration might become more apparent during later stages of the disease [49,50]. Consistent with this, inspection of feature importance revealed that features capturing arm swing were important to distinguish between normal and Parkinsonian gait, while roughness of movement features were important to distinguish between patients with slight, mild and moderate impairment.

We trained a machine learning classifier to distinguish between different UPDRS severities based on these features. Because features are interpretable, the model and its estimates could be inspected in a straightforward manner, and could provide valuable support for assessors. We showed how eccentricity tables and the model’s probability distribution could be used to support a clinician’s decision-making. Feature values for a patient without impairment should generally look most similar to feature values of other patients without impairment. Similarly, feature values of a patient with moderate impairments would be expected to fall most closely to the center of the distribution of other patients with the same level of impairment. Ratings are not always clear-cut, and we showed an example where the model estimate disagreed with the clinician’s rating by one point. The eccentricity table showed less “columnar” structure, with some feature values being typical of healthy gaits, while others were more similar to those of patients with more severe disease. SHAP values provided insight into how the model weighed the importance of these feature values at each step (i.e., within each binary classifier) for the specific example. It was also shown that the estimated probability distribution across severity ratings was considerably flatter than those of the unambiguous examples, highlighting how the model can provide useful information beyond a single most likely estimate.

We selected five videos for which examiner and model scores diverged by two points for re-evaluation by a senior neurologist. The expert agreed with the original assessors’ ratings in only one of these videos. In three cases, the expert’s rating was between the clinician’s and the model’s rating. In one case, the expert agreed with the model rating, disagreeing with the clinician’s rating by two points. Ten additional videos were selected for which the scores of examiners and our model differed by 0 or 1 points. In eight cases, the expert’s ratings matched the original assessor’s rating, half of which also matched the model’s estimates. In two cases, the expert disagreed with the clinician and the model by one point.

### 4.4. Limitations

Several limitations of this study should be noted. Firstly, all gait assessments were performed on patients diagnosed with Parkinson’s disease and our sample therefore did not include healthy control participants. Similar to a previous study [25], we excluded patients with the highest (“severe”) rating on the MDS-UPDRS gait item, due to the small representation of these patients within the dataset. We note that MDS-UPDRS instructions mention that patients should receive a score of 3 (“moderate”) if they require an object to assist, such as a walker or walking stick, but currently our system does not identify any such objects.

Video clips did not show patients walking at an angle substantially away from the coronal plane as examiners asked the patients to walk directly away from and towards the camera. While this is consistent with UPDRS instructions, it is a limitation of the system to have been developed using videos clips that were all recorded at a similar angle.

Our approach relied on manually labelled regions of interest indicating when a patient would start or stop walking towards or away from the camera. This means that, in our analysis, we did not include sections of the video during which patients were turning. Difficulty in turning is another important indicator for PD [59,60,61]. Therefore, it is likely that, for some examples in our data set gait, impairments were primarily evident during turning, leading to misclassifications by the model. Our approach was to focus on a small number of simple and interpretable features, though it is clear they do not capture all of the rich complexities of human gaits [62].

We note that the use of a homogeneous Poisson model, for peak/trough detection when computing step frequency, implies that the probability of a step occurring in any one frame is independent of all other frames and constant across all frames, and that higher step frequency implies a higher variance. However, the probability of a peak/trough occurring at any time is clearly dependent upon the timing of previous peak/troughs. In spite of this assumption, it was clear that estimates were close to the true values that were also contained within the credible intervals in 99.8% of cases.

## 5. Conclusions

### 5.1. Future Work

Further work is needed to establish whether this system could be used to differentiate gait characteristics of healthy controls from those of PD patients, potentially aiding clinicians during (early) diagnosis. Patients are given high ratings (“moderate” or “severe”) if they require assistance in walking (by an object or person, respectively). A system that incorporates the identification of this assistance would likely perform better on these high ratings. Such work would require a dataset with a greater representation of “moderate” or “severe” patients.

Additional work is required to validate the system for different recording methods, such as video clips recorded at 90 degrees from the coronal plane. To that point, 3D models may prove more feature computation more robust to the angle of recording, and so it is possible that 3D reconstruction would improve the performance of this system. Recent studies have shown promise in estimating full 3D pose reconstruction based on data recorded using a single 2D camera [63,64,65,66], although challenges remain [21].

The use of 3D pose estimation may be crucial to addressing other potentially important gait characteristics of PD patients, such as stopped posture [61], left-right asymmetry of arm swing [33], or difficulty in turning [59,60,61]. Furthermore, such a system may potentially not require the need for manually labelled regions of interest, instead using 3D positioning to reliably and automatically label each region of interest (walking towards, turning, walking away).

The use of a non-homogeneous Poisson process to model peak/trough detection when computing step frequency could be investigated, as this would reflect how the probability of peak/trough detection should vary over the course of a gait cycle. Additionally, while the current system only used point estimates of the posterior distributions, future work could consider making use of the estimated uncertainty around it.

### 5.2. Contributions

Quantitative analysis of gait examination has not yet been widely adopted for use by clinicians assessing patients [67], despite readily available technologies enabling this for multiple decades (e.g., accelerometer based systems, [11,68]). Acceptability of new methods rests not only on analytical superiority but also cost and ease of adoption. The approach advanced here relied on videos which are already routinely recorded during examinations at many assessment centers, meaning it did not require alteration of the current gait examination process, during which patients simply walk up and down within an available hallway or some other space.

It is worth emphasising that this means our data were not collected in specialised laboratories or equipment and thus accurately reflect current routine clinical practice. Settings included a number of different rooms, corridors and offices. For this study, videos were collected using an Android app uploaded to a web platform [29]; however, the approach does not rely on any specific device and could be applied to videos recorded using any application or device.

Our approach can support clinicians by providing them with interpretable features. The availability of these objective features in PD has the potential to improve individualised treatments, particularly device based therapies. Deep brain stimulation and infusion therapies may benefit from the ability to titrate against precisely measured motor features.

Automated systems for quantifying Parkinsonian gait have great potential to be used in combination with, or the absence of, trained assessors, during assessments in the clinic or at home. As both clinicians and patients often value explainability, “black box” systems are unlikely to gain widespread adoption. Our approach provides interpretability as well as the ability to trace any (unusual) output back to clinically interpretable features.

### 5.3. Practical Application

In conclusion, we showed that the severity of gait impairment in Parkinson’s disease could be accurately estimated using a single patient video. Data were collected using consumer mobile devices during routine assessments within standard clinical settings. The approach is simple and cheap to implement within existing clinical practice as it does not require any additional setup or equipment, and we showed how the system could support clinicians during routine examinations by providing objective and interpretable estimates. In addition to providing a second objective opinion for gait severity ratings, the model could also be used to estimate an initial rating in the absence of trained assessors—for example, during remote home assessments.

## Figures and Tables

**Table 1:** Summary of signal computation. The first column denotes the symbol used for each signal, the second column gives a brief non-rigorous description of each signal and the third column lists the exact formula used to calculate the value of a signal on frame *t*. $P_{i}^{\left(t\right)}$ denotes the coordinates of body key-point *i* at frame *t*, i.e., a pair of values $\left(x_{i}^{\left(t\right)},y_{i}^{\left(t\right)}\right)$. Relevant key-point indices were neck = 1, right wrist = 4, left wrist = 7, right hip = 9, right ankle = 11, left hip = 12, left ankle = 14, left heel = 21, right heel = 24. *L* and *R* denote the left and right side, respectively, $H^{\left(t\right)}$ denotes the estimated height of the patient (see Supplement), *d* denotes a distance function, *∢* denotes an angle.

Signal	Description	Formula
$R_{legs}\left(t\right)$	$\frac{\mathrm{left}\;\mathrm{leg}}{\mathrm{right}\;\mathrm{leg}}-\frac{\mathrm{right}\;\mathrm{leg}}{\mathrm{left}\;\mathrm{leg}}$	$\frac{|\vec{P_{12}^{\left(t\right)}P_{14}^{\left(t\right)}}|}{|\vec{P_{9}^{\left(t\right)}P_{11}^{\left(t\right)}}|}-\frac{|\vec{P_{9}^{\left(t\right)}P_{11}^{\left(t\right)}}|}{|\vec{P_{12}^{\left(t\right)}P_{14}^{\left(t\right)}}|}$
$A_{body}^{\left[vert\right]}\left(t\right)$	∢(y-axis,d(neck,ankles))	$\sin^{-1}\left(\frac{x_{1}^{\left(t\right)}-x_{11,14}^{\left(t\right)}}{|\vec{P_{1}^{\left(t\right)}P_{11,14}^{\left(t\right)})}|}\right)$
$A_{ankles}^{\left[horiz\right]}\left(t\right)$	∢(x-axis,d(ankle(L),ankle(R)))	$\tan^{-1}\left(\frac{y_{14}^{\left(t\right)}-y_{11}^{\left(t\right)}}{x_{14}^{\left(t\right)}-x_{11}^{\left(t\right)}}\right)$
$A_{wrists}^{\left[horiz\right]}\left(t\right)$	∢(x-axis,d(wrist(L),wrist(R))	$\tan^{-1}\left(\frac{y_{7}^{\left(t\right)}-y_{4}^{\left(t\right)}}{x_{7}^{\left(t\right)}-x_{4}^{\left(t\right)}}\right)$
$D_{heels}^{\left[horiz\right]}\left(t\right)$	$\frac{d(\mathrm{heel}(L),\mathrm{heel}(R))}{H^{\left(t\right)}}$	$\frac{abs(x_{21}^{\left(t\right)}-x_{24}^{\left(t\right)})}{H^{\left(t\right)}}$
$D_{ankle(L)}^{\left[Eucl\right]}\left(t\right)$	$\frac{d({\mathrm{ankle}(L)}^{\left(t\right)},{\mathrm{ankle}(L)}^{\left(t+1\right)})}{H^{\left(t\right)}}$	$\frac{\vec{|P_{14}^{\left(t\right)}-P_{14}^{\left(t+1\right)}|}}{H^{\left(t\right)}}$
$D_{ankle(R)}^{\left[Eucl\right]}\left(t\right)$	$\frac{d({\mathrm{ankle}(R)}^{\left(t\right)},{\mathrm{ankle}(R)}^{\left(t+1\right)})}{H^{\left(t\right)}}$	$\frac{\vec{|P_{11}^{\left(t\right)}-P_{11}^{\left(t+1\right)}|}}{H^{\left(t\right)}}$

**Table 2:** Association between feature values and total UPDRS part-III scores. The postural control feature showed the highest correlation with total UPDRS part-III (Pearson’s r=−0.31, p<0.001).

Feature	Pearson’s *r*	*p*-Value
Speed	−0.26	<0.001
Arm swing (velocity)	−0.31	<0.001
Arm swing (amplitude)	−0.27	<0.001
Postural control	−0.31	<0.001
Roughness (min)	0.15	<0.001
Roughness (max)	0.13	<0.001

**Table 3:** Results of Mann–Whitney U tests [45] for the difference between feature values from `on medication’ and `off medication’ assessments, alongside the probability of this difference being greater than zero and the corresponding binomial test (two-sides test for this probability differing from 0.5).

Feature	Mann–Whitney’s *U*	*p*-Value	P (Diff > 0)	*p*-Value
Speed	6144	0.001	0.64	0.002
Arm swing (velocity)	4776	<0.001	0.75	<0.001
Arm swing (amplitude)	4965	<0.001	0.77	<0.001
Postural control	6908	0.038	0.58	0.090
Roughness (min)	6303	0.002	0.35	<0.001
Roughness (max)	6658	0.014	0.33	<0.001

**Table 4:** Summary of classification metrics for the six types of models. RFC, Random Forest Classifier; LDA, Linear Discriminant Analysis; LOGIS, Logistic Regression; ANN, Artificial Neural Network; SVM, Support Vector Machine; XGBoost, Gradient Boosted Trees. The RFC was picked as it gave the best performance on three of the four classification metrics.

	Accuracy	Balanced Accuracy	Accuracy (±1)	Spearman’s ρ
RFC	**0.50**	0.50	**0.95**	**0.52**
LDA	0.48	0.51	0.93	0.47
LOGIS	0.45	0.50	0.92	0.47
ANN	0.46	0.41	0.92	0.32
SVM	0.46	**0.52**	0.93	0.49
XGBoost	0.47	0.49	0.93	0.50

**Table 5:** Fifteen videos were sent to a senior neurologist for re-rating: Five videos for which the model estimation disagreed with the original examiner’s rating by 2, and ten videos for which the residual was 1 or 0 points. Each column shows the three different ratings (original examiner, model and expert) for a video, with color shading indicating the level of absolute residual (red = 2, yellow = 1, green = 0).

	Residuals = 2					Residuals = 1				Residuals = 0					
Original Clinical UPDRS	2	1	2	0	2	1	2	2	0	2	0	3	1	2	1
Re-rated Clinical UPDRS	0	2	1	1	2	1	2	2	0	2	0	3	1	1	0
Model Estimated UPDRS	0	3	0	2	0	2	1	1	1	2	0	3	1	2	1

## Data Availability

Restrictions apply to the availability of these data. Data were collected at the Department of Clinical and Movement Neurosciences (Institute of Neurology, University College London), Dementia Research Centre (Institute of Neurology, University College London), Neuroscience Research Centre (Molecular and Clinical Sciences Research Institute, St George’s, University of London), Parkinson’s Disease and Movement Disorders Center (Baylor College of Medicine), and The Starr Lab (University of California San Francisco). Current agreements do not allow us to make this data publicly available.
